# Organ-Specific Determinants of Tolerance and the Unique Challenge of Vascularized Composite Allotransplantation

**DOI:** 10.3389/ti.2025.16017

**Published:** 2026-01-14

**Authors:** Haizam Oubari, Loïc Van Dieren, Curtis L. Cetrulo, Alexandre G. Lellouch

**Affiliations:** 1 Plastic Surgery Research, Massachusetts General Hospital Center for Transplantation Sciences, Charlestown, MA, United States; 2 Harvard Medical School, Boston, MA, United States; 3 Chirurgie Plastique, Hopital de la Croix-Rousse, Lyon, France; 4 Cedars-Sinai Medical Center, Los Angeles, CA, United States

**Keywords:** animal models, chimerism, organ specific tolerance, tolerance induction, Vascularized Composite Allotransplantation

We read with great interest the article Tolerance Induction Strategies in Organ Transplantation: Current Status and Future Perspectives. Transplant International. 2025; 38. [1], by Blein T, Ayas N, Charbonnier S, Gil A, Leon J, Zuber J., which offers a timely and well-organized synthesis of the major tolerance-induction strategies across transplantation. Presenting these diverse approaches, ranging from mixed hematopoietic chimerism to regulatory–cell–based interventions, within a single conceptual framework is valuable for the field, particularly as tolerance research continues to evolve at the intersection of immunology, engineering, and translational science. The authors should be commended for their clear exposition and for stimulating a broader discussion on how these pathways could be accurately integrated into future clinical applications, particularly given that, to date, the only reliably effective approach to tolerance induction has been the chimerism-based strategy. They also appropriately underscore the crucial issue of model selection: large animals, especially nonhuman primates (NHPs) or wild-caught species, more reflect the clinical complexity of tolerance induction than environmentally controlled, antigen-limited laboratory mice, as the latter lack the far richer and more heterogeneous repertoire of potentially alloreactive memory T cells typically present in NHPs and human recipients [2]. These models are increasingly challenging to implement, owing to rapidly escalating costs and heightened regulatory scrutiny aimed at ensuring ethical research. Nevertheless, they remain indispensable for meaningful translational progress. In the spirit of expanding this conversation, we believe it is important to highlight an additional dimension that can profoundly influence the success of tolerance-induction strategies: the organ-specific nature of antigenicity and immunologic permissiveness, briefly addressed in this review. These distinctions have significant implications for the application of chimerism-based and cellular therapies across various graft types.

Preclinical and clinical data demonstrate that solid organs differ markedly in their intrinsic antigenicity, inflammatory profiles, and thresholds for tolerogenic conditioning. Intra-abdominal organs such as the kidneys and liver are inherently more permissive to tolerance, whereas hearts and lungs remain tolerance-resistant. These organ-specific disparities help understand why chimerism-based protocols that reliably induce renal tolerance often fail in thoracic organs, underscoring the necessity of interpreting tolerance strategies through an organ-specific rather than organ-agnostic lens [3]. For instance, kidneys are consistently the most amenable organs for tolerance induction: in both NHP models and haplomatched human recipients, mixed hematopoietic chimerism, often transient, has been sufficient to achieve long-term, immunosuppression-free renal allograft survival [4–6]. In fact, kidneys are often considered to possess a protolerogenic potential, a concept further supported by recent MGH findings showing kidney-induced cardiac allograft tolerance in the NHP model [7]. In striking contrast, other solid organs such as the heart [8] and lung grafts remain considerably more refractory, necessitating often stronger immunosuppressive regimens. Cardiac and pulmonary grafts exhibit heightened ischemia–reperfusion injury, stronger innate immune activation, and more proinflammatory tissue-resident leukocyte compartments. These features drive accelerated effector priming, stronger indirect allorecognition, and a limited capacity to sustain donor hematopoietic engraftment, making these organs disproportionately resistant to both chimerism-based and regulatory-cell–based strategies [3]. These mechanistic observations underscore that tolerance induction is fundamentally shaped by organ-intrinsic biology, with mixed chimerism proving far more stable and effective in kidneys and liver than in thoracic organs.

These disparities become even more pronounced when considering vascularized composite allotransplantation (VCA). High immunosuppressive requirements have, to date, drastically limited the number of VCA procedures performed worldwide [9], and this translates into a particular complexity when applying tolerance-induction strategies to these grafts. VCAs contain multiple, highly antigenic, leukocyte-rich tissues, including skin and mucosa, as well as ischemia-sensitive components such as muscle. This places them at the extreme end of the tolerance-resistance spectrum. In swine, transient mixed chimerism is insufficient to induce full VCA tolerance, and a characteristic split-rejection phenomenon, marked by acceptance of musculoskeletal elements but rejection of the skin, has been consistently observed [10]. Achieving stable, multilineage chimerism is required for tolerance of all VCA components; this has only been accomplished through intensified conditioning regimens incorporating augmented irradiation, CTLA4-Ig, anti-IL-6R therapy, and vascularized bone marrow, which enabled long-term tolerance of skin-bearing VCAs across class-I barriers in a clinically relevant model [11]. Nonhuman primate data further underscore this divide: prior delayed-tolerance induction protocols in cynomolgus macaques generated robust renal tolerance under identical conditioning yet consistently failed in hand or face VCA models, with early rejection, infectious complications, and absence of chimerism [12]. More recently, our group demonstrated, for the first time in the NHP partial face transplant model, that simultaneous tolerance induction can generate transient myeloid and lymphoid chimerism, allowing for prolonged immunosuppression-free survival of a face allograft, although the graft ultimately underwent split and then full rejection [13]. Collectively, these findings highlight that VCA immunobiology differs substantially from that of solid organs, cautioning against the direct extrapolation of kidney-derived tolerance strategies to the multi-tissue context of VCA. Furthermore, the extreme sensitivity of these grafts to ischemia–reperfusion injury suggests that they may substantially benefit from *ex vivo* preservation, preconditioning and reengineering strategies [14], as also highlighted by Blein et al.

Taken together, these organ- and species-specific distinctions, further magnified in VCA, underscore that tolerance strategies cannot simply be transferred from one graft type to another. They also outline multiple conceptual layers that shape tolerance-induction research and its clinical translation (Figure 1). Against this backdrop, the authors’ effort to synthesize cross-organ tolerance mechanisms and to delineate shared versus organ-specific barriers is both timely and necessary, and their work represents a highly relevant contribution to the field.

**FIGURE 1:**
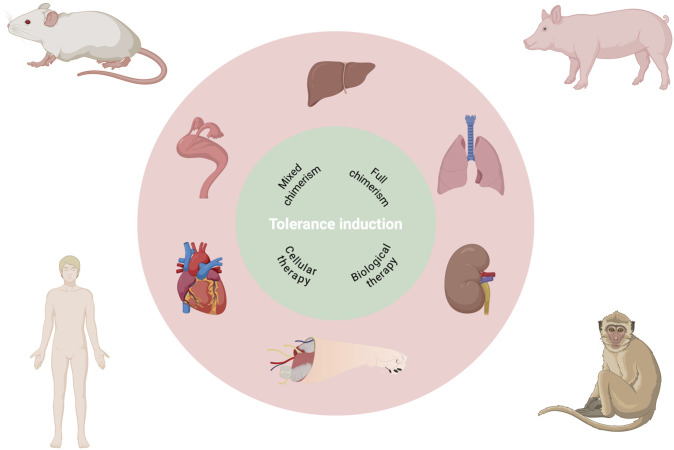
Multilayered complexity for translational research in allotransplantation tolerance.

## Data Availability

The original contributions presented in the study are included in the article/supplementary material, further inquiries can be directed to the corresponding author.

## References

[1] Blein T Ayas N Charbonnier S Gil A Leon J Zuber J . Tolerance Induction Strategies in Organ Transplantation: Current Status and Future Perspectives. Transpl Int (2025) 38:14958. 10.3389/ti.2025.14958 41127478 PMC12539449

[2] Nadazdin O Boskovic S Murakami T O'Connor DH Wiseman RW Karl JA Phenotype, Distribution and Alloreactive Properties of Memory T Cells from Cynomolgus Monkeys. Am J Transplant (2010) 10(6):1375–84. 10.1111/j.1600-6143.2010.03119.x 20486921 PMC2893326

[3] Hull TD Benichou G Madsen JC . Why Some Organ Allografts are Tolerated Better than Others: New Insights for an Old Question. Curr Opin Organ Transplant (2019) 24(1):49–57. 10.1097/MOT.0000000000000594 30516578 PMC6467256

[4] Kawai T Sachs DH Sykes M Cosimi AB , Immune Tolerance Network. HLA-Mismatched Renal Transplantation Without Maintenance Immunosuppression. New Engl J Med (2013) 368(19):1850–2. 10.1056/NEJMc1213779 23656665 PMC3760499

[5] Kawai T Sachs DH Sprangers B Spitzer TR Saidman SL Zorn E Long-Term Results in Recipients of Combined HLA-Mismatched Kidney and Bone Marrow Transplantation Without Maintenance Immunosuppression. Am J Transplant (2014) 14(7):1599–611. 10.1111/ajt.12731 24903438 PMC4228952

[6] Sasaki H Hirose T Oura T Otsuka R Rosales I Ma D Selective Bcl-2 Inhibition Promotes Hematopoietic Chimerism and Allograft Tolerance Without Myelosuppression in Nonhuman Primates. Sci Translational Med (2023) 15(690):eadd5318. 10.1126/scitranslmed.add5318 37018417 PMC11022838

[7] Tonsho M O JM Ahrens K Robinson K Sommer W Boskovic S Cardiac Allograft Tolerance Can Be Achieved in Nonhuman Primates by Donor Bone Marrow and Kidney Cotransplantation. Sci Translational Med (2025) 17(782):eads0255. 10.1126/scitranslmed.ads0255 39841809 PMC12186763

[8] Chaban R Ileka I Kinoshita K McGrath G Habibabady Z Ma M Enhanced Costimulation Blockade with αCD154, αCD2, and αCD28 to Promote Heart Allograft Tolerance in Nonhuman Primates. Transplantation (2025) 109(6):e287–e96. 10.1097/TP.0000000000005315 39792548 PMC12097961

[9] Van Dieren L Tawa P Coppens M Naenen L Dogan O Quisenaerts T Acute Rejection Rates in Vascularized Composite Allografts: A Systematic Review of Case Reports. J Surg Res (2024) 298:137–48. 10.1016/j.jss.2024.02.019 38603944

[10] Mathes DW Randolph MA Solari MG Nazzal JA Nielsen GP Arn JS Split Tolerance to a Composite Tissue Allograft in a Swine Model. Transplantation (2003) 75(1):25–31. 10.1097/00007890-200301150-00005 12544866

[11] Lellouch AG Andrews AR Saviane G Ng ZY Schol IM Goutard M Tolerance of a Vascularized Composite Allograft Achieved in MHC Class-I-Mismatch Swine via Mixed Chimerism. Front Immunol (2022) 13:829406. 10.3389/fimmu.2022.829406 35619720 PMC9128064

[12] Lellouch AG Ng ZY Rosales IA Schol IM Leonard DA Gama A-R Toward Development of the Delayed Tolerance Induction Protocol for Vascularized Composite Allografts in Nonhuman Primates. Plast and Reconstr Surg (2020) 145(4):757e–68e. 10.1097/PRS.0000000000006676 32221215

[13] Oubari H Van Dieren L Lancia H Dehnadi A Jeljeli M Randolph M Long Term Immunosuppression Free Survival of a Face Allotransplant via Donor Hematopoietic Chimerism in Non Human Primates. Am J Transplant (2025) 25(8):S535. 10.1016/j.ajt.2025.07.1239

[14] Oubari H Van Dieren L Berkane Y Jeljeli M Randolph MA Uygun K 30. *Ex vivo* Preservation and Study of a Non-Human Primate Partial Face Transplant Model Using Sub Normothermic Machine Perfusion. Transplantation (2025) 109(6S2):19. 10.1097/01.tp.0001123868.81813.0b

